# Anticarcinogenic cationic peptides derived from tryptic hydrolysis of β-lactoglobulin

**DOI:** 10.3389/fmolb.2024.1444457

**Published:** 2025-01-07

**Authors:** Eman Ibrahem, Ali Osman, Hefnawy Taha, Mohamed F. Abo El-Maati, Basel Sitohy, Mahmoud Sitohy

**Affiliations:** ^1^ Department of Biochemistry, Faculty of Agriculture, Zagazig University, Zagazig, Egypt; ^2^ Department of Clinical Microbiology, Infection and Immunology, Umeå University, Umeå, Sweden; ^3^ Department of Diagnostics and Intervention, Oncology, Umeå University, Umeå, Sweden

**Keywords:** β-lactoglobulin, trypsin, antioxidant activity, anticancer activity, caspase, VEGFR-2

## Abstract

**Introduction:**

This study investigated the tryptic hydrolysis of β-lactoglobulin (BLG) for 30, 60, 90, and 120 min at 1/200 E/S (enzyme/substrate ratio, w/w) to prepare potentially anticarcinogenic peptides.

**Methods:**

The properties of hydrolysates were characterized, including degree of hydrolysis, free amino acids, SDS-PAGE, FTIR, and antioxidant activity employing DPPH-assay, β-carotene/linoleic acid, and FRAP assay.

**Results:**

BLG tryptic hydrolysate produced after 60 min hydrolysis recorded the highest antioxidant activity, and LCMS analysis revealed 162 peptides of molecular masses ranging from 800 to 5671Da, most of them are of hydrophobic nature. Within the low-MW peptide group (24 peptides), there were nine hydrophobic basic (HB) and seven hydrophobic acidic (HA), representing 38% and 29%, respectively. The HB peptides may be responsible for the considerable biological activity of the hydrolysate. With dominant basic character supporting the carcinogenic activity of this hydrolysate. The *in vitro* anticancer activity against Mcf-7, Caco-2, and A-549 human cancer cell lines proliferation by MTT assay recorded IC_50%_ at 42.8, 76.92, and 45.93 μg/mL, respectively. Treating each cell line with IC_50%_ of the hydrolysate for 24 h increased the apoptosis by enhancing the expression of caspase-9 by 5.66, 7.97, and 3.28 folds over the untreated control and inhibited angiogenesis by reducing VEGFR-2 expression by about 56, 76, and 70%, respectively, indicating strong anticancer and antiangiogenic actions on human cancer cells. BLG tryptic hydrolysate may serve as a natural anticarcinogenic agent. The results of this study demonstrated that BLG hydrolysates have direct anticancer and antiangiogenic effects on human cancer cells. The chemical composition and characteristics of the BLG tryptic hydrolysate influence these biological and anticancer activities. The tryptic hydrolysates were generally effective against the three cancer cell lines studied (Mcf-7, Caco-2, and A-549). This effectiveness was assessed by measuring cell proliferation using the MTT assay and by evaluating their impact on angiogenesis through inhibition of VEGFR-2 activity.

**Discussion:**

Future studies may focus on enhancing the anticarcinogenic effectiveness of the peptides by isolating and evaluating the most prominent individual peptide and varying the treatment conditions.

## 1 Introduction

The oxidative stress engendered by the reactive oxygen species engenders significantly affects several age-associated diseases, neurodegenerative disorders, and cancer development (Cui et al., 2004; Abdel-Hamid et al., 2020a; Imbabi et al., 2021a). Hence, oxidation processes in the body and foodstuffs have been the focus of several investigations. Oxidative reactions may trigger food quality deterioration, unacceptable taste and color, loss of nutritive value, and reduced shelf life. Lipid peroxides and low molecular weight compounds produced during post-oxidative reactions may produce lethal cellular effects. Lipids, proteins, DNA, and enzymes are the main targets of oxidation reactions. Preventing food deterioration may protect against the processes promoting the development of age-linked diseases, such as cancer, atherosclerosis, and diabetes (Wang et al., 2022; Sheng et al., 2023; Zheng et al., 2023). Hence, it is essential to control the lipid peroxidation in foodstuffs and the body (Nwachukwu and Aluko, 2019; Ramkisson et al., 2020; Imbabi et al., 2021b). The potential adverse hazards of artificial antioxidants (e.g., butylated hydroxy anisole and butylated hydroxytoluene) have considerably prompted the search for natural antioxidants (Imbabi et al., 2021a; Wang et al., 2021; Kong et al., 2023).

Cancer has globally become a severe health concern; WHO is estimating 13.1 million cancer-associated deaths in 2030 (Ramkisson et al., 2020). However, current epidemiological studies indicated a decreased dominance of chronic diseases and cancer consistently associated with consuming certain nutrients. As a result, functional foods may be suggested as complementary agents in cancer treatment.

Reactive oxygen species (ROS), e.g., hydroxyl free radicals, hydrogen peroxide, oxygen singlet, and superoxide anions, are usually triggered by endogenous and exogenous stimuli. Living organisms often neutralize them using well-established endogenous antioxidant defense systems (Nwachukwu and Aluko, 2019). However, ROS can disrupt natural defense systems when produced in excess, enhancing oxidative stress. Sustainably cumulative oxidative stress may incur harmful oxidative injuries to biological macromolecules such as proteins, lipids, and nucleic acids leading to irreversible alterations in cellular functions (Nwachukwu et al., 2019). Oxidative damage can seriously affect DNA structure and functionality. ROS can interact with biological constituents, e.g., phospholipids and proteins, forming secondary reactive intermediates, which can irreversibly bind to DNA bases, forming DNA adducts. Consequently, DNA adducts may evade cellular repair mechanisms and promote miscoding and even mutations, paving the way for carcinogenic processes (Fuchs-Tarlovsky, 2013). Studies have related oxidative stress to the pathogenesis of inflammatory cancers. Thus, agents capable of protecting cells against ROS attack via quenching free radicals are considered potent chemo-preventive candidates.

The synthesis of reactive oxygen species (ROS) may result in oxidative stress and many conditions, such as cancer (Ho et al., 2013). Oxidative stress markers were significantly elevated in patients with pancreatic cancer compared to the controls (Kodydkova et al., 2013). Furthermore, relative to healthy cells, cancer cells exhibit significantly increased steady-state levels of ROS (Aykin-Burns et al., 2009). There is a substantial increase in intracellular O_2_ and H_2_O_2_ in cancer cell mitochondria related to normal cells and this could designate a purpose for improving cancer therapy (Aykin-Burns et al., 2009). Due to their fast metabolism and high proliferation rate, cancer cells produce a higher concentration of ROS. Based on the close interaction between ROS, platelets, and cancer ROS was concluded to play a stimulative role in tumor growth and metastasis through platelets (Zhang et al., 2023). Breast cancer was reported to stimulate oxidative stress responses, producing different inflammatory cytokines and pronouncing the relationship between oxidative stress parameters and these cytokines, which are highly associated with aggressive tumors (Ghafoor, 2023). A correlation between antioxidative capacity and anticancer efficiency has been established in a variety of biological and chemical agents, encompassing plant phenolics and dietary kelp (Dia and de Mejia, 2010).

At low to moderate levels, ROS may contribute to tumor formation by acting as signaling molecules or by promoting the mutation of genomic DNA. For instance, ROS can stimulate the phosphorylation of mitogen-activated protein kinase (MAPK) and extracellular signal-regulated kinase (ERK), cyclin D1 expression and JUN N-terminal kinase (JNK) activation, all of which are linked to tumor cell growth and survival (Martindale and Holbrook, 2002; Ranjan et al., 2006). At high levels, ROS promotes cell death and severe cellular damage. Cancer cells need to combat high levels of ROS, especially at the early stages of tumor development. Research has revealed that conditions that induce oxidative stress also increase the selective pressure on pre-neoplastic cells to develop powerful antioxidant mechanisms. High ROS levels are also induced by detachment from the cell matrix (Schafer et al., 2009). This aspect represents a challenge for metastatic cancer cells that need to survive during migration to distant organs (Gorrini et al., 2013).

One practical approach to overcome these hurdles is using antioxidants and anticancer peptides from natural sources. Recently, a growing interest in the role of bioactive peptides derived from food proteins has emerged. Bioactive peptides are biomolecules enzymatically released from proteins, usually containing 2–20 amino acids, possessing significant biological properties, variating from antioxidant, anti-diabetic and anticancer activities (Islam et al., 2022). Additionally, peptides and amines were involved in the pathogenesis and treatment of colon cancer (Sitohy and El-Salhy, 2001; El-Salhy and Sitohy, 2002; Sitohy and El-Salhy, 2002; Sitohy and El-Salhy, 2003; El-Salhy et al., 2003; Ali et al., 2021). Others could serve as prognostic biomarkers (Rashad et al., 2018; AbdelMageed et al., 2019; Ali et al., 2019). The dairy industry is based on transforming raw milk into food products encompassing butter, cheese, yogurt, ice cream, and desserts. Many by-products emitting high organic matter from this industry, e.g., carbohydrates, proteins, lipids, and minerals, incur environmental pollution. Whey is a significant component of the dairy industry, highly rich in proteins and lactose. Whey proteins (WP) include ca. 40%–50% β-lactoglobulin (β-Lg), ca. 12%–15% α-lactalbumin (α-lac), and ca. 5% bovine serum albumin (BSA) (Yadav et al., 2015). Studies targeting the peptic or tryptic hydrolysis of whey protein components indicated the generation of peptides with new functionalities (AbdelMageed et al., 2019; Ali et al., 2021). The protein-derived fragments providing human health benefits are termed bioactive peptides (Bhandari et al., 2020). In recent years, several studies extracted bioactive peptides from food proteins and explored their numerous health benefits (Abdel-Hamid et al., 2016; Abdel-Hamid et al., 2017; Abdel-Hamid et al., 2020b; Osman, et al., 2021a). These bioactive peptides are naturally produced by enzymatic proteolysis during gastrointestinal digestion. Alternatively, they can be *in-vitro* released by protein hydrolysis with some food-grade proteolytic enzymes and during food processing (cooking, ripening, and fermentation) (Osman et al., 2016; El-Sanatawy et al., 2021; Osman et al., 2021b; Osman et al., 2021c). Milk proteins were studied for possible degradation and revealing different information on the functionality of the issued peptides (Chobert et al., 1987; Chobert et al., 1988; Chobert et al., 2005; Abdel-Hamid et al., 2020a). Newly discovered peptides exhibited multiple bioactivities triggered by their antioxidant, antihypertensive, and anticancer traits (Chi et al., 2015; Qiao et al., 2022). Antioxidant peptides are prominently contributing to the management of non-communicable degenerative diseases, such as cancer, rheumatoid arthritis, diabetes, or cardiovascular and cerebrovascular diseases (Zou et al., 2016). The most attractive advantage of natural peptides is their minimal side effects in humans.

β-Lactoglobulin is particularly suitable for deriving anticancer peptides, being rich in sequences that can, upon enzymatic hydrolysis, release bioactive peptides with various biological activities, including anticancer, and exhibiting strong antioxidant activities, which participate in counteracting cancer development (Hernández-Ledesma et al., 2008). Additionally, some peptides released from β-lactoglobulin upon enzymatic hydrolysis were confirmed to induce apoptosis in carcinogenic cells as a crucial means of removing cancer cells and at the same modulate the immune system, enhancing the body’s ability to fight cancer cells, finally leading to reduced rates of cancer cell proliferation (Ghadiri et al., 2024). So, considering all these facts makes β-lactoglobulin a valuable source for exploring new bioactive peptides β-lactoglobulin with potential anticancer action. Therefore, the objectives of this study were to (i) investigate the short-time tryptic hydrolysis of β-lactoglobulin, (ii) estimate the antioxidant and anticancer bioactivities of the resulting peptides, and (iii) identify the peptides potentially responsible for the recorded bioactivities.

## 2 Materials and methods

### 2.1 Materials and reagents

β-Lactoglobulin from bovine milk, and Trypsin from porcine pancreas with specific activity 1,000–2,000 BAEE units/mg solid (EC3.4.21.4) were obtained from Merck (KGaA, Darmstadt, Germany). All the reagents and chemicals obtained from commercial sources were of analytical grade.

### 2.2 Preparation and characterization of protein hydrolysates

#### 2.2.1 β-Lactoglobulin hydrolysates preparation

Enzymatic hydrolysis of β-lactoglobulin was conducted using trypsin under the following conditions: buffer, 0.1 M Na_2_HPO_4_–NaH_2_PO_4_; pH, 8.8; temperature, 37°C and 1/200 E/S (w/w) (enzyme/substrate ratio). The substrate and enzyme were thoroughly mixed and incubated under continuous stirring at 37°C for 120 min. Then, the enzyme was inactivated by heating the mixture in a boiling water bath at 100°C for 10 min. Finally, the hydrolysate was centrifuged at 4,000 *g* and 4°C for 10 min. The supernatant was lyophilized and kept at −20°C until use (Abdel-Hamid et al., 2017).

#### 2.2.2 Degree of hydrolysis

The degree of hydrolysis was assessed using the trichloroacetic acid (TCA) method (Hoyle and Merrltt, 1994). Following hydrolysis, 1 mL of protein hydrolysate was combined with 1 mL of 20% (w/v) TCA to create 10% TCA soluble material. The mixtures were allowed to stand for 30 min to enable precipitation, then centrifuged at 8,000 *g* for 10 min. The liquid portion containing soluble nitrogen in TCA 10% was freeze-dried and examined for protein levels using the Kjeldahl method. The initial sample (protein hydrolysates) was assessed for protein content using the Kjeldahl method. The degree of hydrolysis (DH) was determined using the following formula:

$$Degree\;of\;hydrolysis\;\left(\%\right)=\left(Soluble\;nitrogen\;in\;TCA\;10\%/\;Total\;nitrogen\;in\;the\;sample\right)\;x\;100$$

#### 2.2.3 Free amino acids estimation

Free amino acids were estimated using the ninhydrin method after 30, 60, 90, and 120 min-hydrolysis (Lie, 1973). Two mL of diluted samples (1–3 mg/L) were mixed with 1 mL of the ninhydrin solution (0.1 M in phosphate buffer, pH 6.7) and boiled in a boiling water bath for 20 min before cooling for 20 min in a cold-water bath (4°C). The cooled mixture was combined with 5 mL of 0.2% potassium iodide solution (in 60% ethanol), and the developed color was measured at 570 nm. The calibration equation for leucine X = (y-0.1499)/0.0011 (R2 = 0.9455), where x is leucine concentration in µg/mL and y is leucine absorbance.

#### 2.2.4 SDS-polyacrylamide gel electrophoresis (SDS-PAGE)

SDS–PAGE of β-lactoglobulin tryptic hydrolysates at different times (0, 30, 60, 90, and 120 min) was run according to Laemmli (1970), using 3% stacking acrylamide gel and 17% resolving acrylamide gel.

#### 2.2.5 FTIR spectroscopy (fourier transform infrared)

The structural conformation and the functional groups of β-lactoglobulin hydrolysate were analysed by the FTIR technique (Rosli and Sarbon, 2015). An amount (1.0 mg) of the dried hydrolysate was added to approximately 100 mg of potassium bromide (KBr) and finely ground. The resulting powder was placed in a palletizer, forming a miniature thin disc, which was subsequently, located in a Thermo Nicolet 380 Spectrometer (Fisher Scientific Inc., United States). The emitting spectra were recorded at wavenumber from 4,000 to 500 cm-1 and at a data acquisition rate of 2 cm-1 per point. The background was deduced from the data using Opus software (Fisher Scientific Inc., United States).

#### 2.2.6 Nano-LC MS/MS analysis

The 60-min tryptic protein hydrolysate, with the highest antioxidant activity, was subjected to nano-LC MS/MS analysis in the positive ion mode. This analysis was performed using a Triple TOF 5600 +(AB Sciex, Canada) connected with an Eksigent nano-LC 400 autosampler with an Ekspert nanoLC 425 pump at the front end. Peptides were trapped in the trap and elute mode on a CHROMXP C18CL 5 mm column (10 0.5 mm) (Sciex, Germany). MS and MS/MS ranges were 400–1,250 m/z and 170–1,500 m/z, respectively. Samples were eluted on a 55-min linear gradient 3%–40% solution (80% ACN, 0.2% formic acid). The 40 most intense ions were sequentially selected in data-dependent acquisition (DDA) mode with a 2–5 charge state. Surveys of full scan MS and MS/MS spectra were estimated at resolutions of 35.000 and 15.000, respectively. External calibration was scheduled and run during sample batches to correct possible TOF deviation and ensure the accuracy of the analysis (Saadeldin et al., 2020).

### 2.3 Antioxidant activity evaluation

The antioxidant activity of β-lactoglobulin tryptic hydrolysates (1,000 μg/mL) obtained at different periods (0, 30, 60, 90, and 120 min) was estimated using several methods such as DPPH radical scavenging activity, β-carotene/linoleic acid bleaching, and ferric reducing antioxidant power. The BLG -60 min tryptic hydrolysate was selected for this analysis at different concentrations (50–1,000 μg/mL).

#### 2.3.1 DPPH radical scavenging activity assay

The DPPH radical scavenging activity was determined following (Ramadan et al., 2008; Gocer and Gulcin, 2011) with some modification. 1 mL of each sample at different concentrations (50–1,000 μg/mL) was combined with 4 mL of 0.15 mM DPPH (in 95% ethanol) and vigorously vortexed. The reaction mixture was incubated in darkness at room temperature for 30 min before measuring the absorbance at 517 nm. TBHQ served as a standard sample. The radical scavenging capacity of the samples was estimated as the decrease in the DPPH radicals’ absorbance and calculated using the equation:

$$Radical\;scavenging\;activity\;\left(\%\right)=\left[\left(A\;control-A\;sample\right)/A\;control\right]\,x\;100$$

A = absorbance at 517 nm.

#### 2.3.2 β-carotene/linoleic acid bleaching method

The capacity of β-lactoglobulin hydrolysates to inhibit the bleaching of β-carotene was examined as reported by (Dastmalchi et al., 2007), using TBHQ as a standard. An amount (0.2 mg) of β -carotene in 1 mL chloroform, 20 mg of linoleic acid, and 200 mg of Tween 20 were well mixed in a round-bottom flask. After removing the chloroform, 50 mL of distilled water was added, and the mixture was vigorously stirred. Three mL of emulsion aliquots were distributed in tubes containing either the tested samples or TBHQ. After mixing, 0.5 mL of each sample was pipetted to a cuvette, and the absorbance was recorded at 470 nm. The remaining samples were placed in a 50°C-water bath for 120 min; then, the absorbance was recorded at 470 nm. Finally, a control without an added sample or TBHQ was assessed. The protection index (PI) was calculated following the equation:

Protection index (%) = A/A0 × 100

Wherein A0 sample is the absorbance of the sample at zero-time, A is the absorbance after 120 min.

#### 2.3.3 Ferric reducing antioxidant power (FRAP) method

The reducing power of β-lactoglobulin hydrolysates and TBHQ was estimated by measuring the absorption of Perl’s Prussian blue complex resulting from reducing Fe^3+^ to Fe^2+^ at 700 nm, following (Gocer and Gulcin, 2011).

### 2.4 Anticancer activity evaluation

#### 2.4.1 Cell viability *in vitro* (MTT-assay)

The impact of BLG 60-min tryptic hydrolysate, at a 31.25–1,000 μg/mL concentration range, on human cancer cell lines, MCF7 (Michigan Cancer Foundation-7), A549 (adenocarcinoma human alveolar basal epithelial cells), Caco-2 (human colorectal adenocarcinoma cells), and normal cell line (FSU, primary foreskin fibroblast cells), viability was assessed *in vitro* using MTT assay (Pan et al., 2016; Pan et al., 2016). The 4 cell lines were grown in Dulbecco’s modified Eagle’s medium (DMEM, Sigma-Aldrich) supplemented with 10% heat-inactivated fetal bovine serum (FBS), penicillin (10 U/mL, Sigma-Aldrich), and streptomycin (10 g/mL, Sigma-Aldrich). The cultures were incubated at 37°C, 5% CO_2_, and 100% relative humidity. The cells were inoculated in 96-well microplates at 10 × 10^3^ cells/well density and grown for 24 h at 37°C in 5% CO_2_ before adding the tested samples. The cells were treated with various concentrations (31.25–1,000 μg/mL) of BLG 60-min tryptic hydrolysate dissolved in phosphate-buffered saline (PBS). Cell viability was determined using the colorimetric MTT assay (Promega, Madison, WI, United States) after 48 h-incubation by measuring the absorbance at 550 nm (Hansen et al., 1989). A definite aliquot (10 µL of a 10% solution) of Triton X-100 was used as the positive control, whereas untreated cells were used as the negative control. The following formulas calculated the percentage of cell viability and cytotoxicity:

$$Cell\;viability\;\left(\%\right)=\left(Ab\;sample/Ab\;control\right)x\;100.$$

Cytotoxic activity (%) of the tested substance was calculated following the formula:

$$Cytotoxic\;activity\;\left(\%\right)=100\;\%-cell\;viability\;\left(\%\right).$$

The hydrolysate concentration producing 50% growth inhibition is termed IC_50_.

#### 2.4.2 Quantification of mRNA levels of Caspase-9

Step-One Plus Real-time PCR (Applied Biosystems, Foster City, CA, United States of America) was employed to quantitatively analyze caspase-9 in three human cancer cell lines (Mcf-7, Caco-2, and A-549), before and after treatments, using gene-specific primers and SYBR Green master mix. The Oligo seven software designed the primers, and they were all tested for spasticity and accuracy on the NCBI website. Cells were treated with IC50 of the tested hydrolysates for each cell line for 24 h. The relative expression level of caspase-9 in mRNA levels was estimated by quantitative real-time PCR. For each sample, the average score of duplicated Ct values was measured, and the comparative Ct method was used to determine the target genes’ relative expression levels. The primer sequences were 5ʹ-GCAGGCTCTGGATCTCGGC-3ʹ and 5ʹ-GCTGCTTGCCTGTTAGTTCGC-3ʹ for the Caspase 9-forward and Caspase 9-reverse with annealing temperature 60.5 and 59.5^ᵒ^C, respectively (Asadi et al., 2018).

#### 2.4.3 *In vitro* VEGFR-2 kinase assay

The most active antiproliferative concentrations of BLG 60-min tryptic hydrolysate (42.8, 76.92, and 45.93 μg/mL) against Mcf-7, Caco-2, and A-549, respectively, were selected for evaluating their inhibitory activities against VEGFR-2 following the manufacturer’s instructions (Abou-Seri et al., 2016) as described in (El-Helby et al., 2019).

The 3 cell lines (5 × 10^5^ cells per well, six wells per plate) were incubated overnight in culture. The serum-free culture-conditioned medium replaced the medium. BLG tryptic hydrolysate was added to the culture, and the medium was collected after 72 h incubation (Garvin et al., 2005). The levels of VEGFR-2 were assessed using a VEGF enzyme-linked immunosorbent assay (ELISA) kit (DVE00, R&D Systems, Minneapolis, MN, United States of America) following the manufacturer’s instructions. Each well’s optical density at 570 nm was measured using an automated microplate reader (model 550, Bio-Rad, Hercules, CA, United States of America). Comparing the treated compounds to the control incubations yielded the inhibition percentage.

$$\%\;Inhibition=\left[\left(control-treatment\right)/\;control\;x\;100\right]$$

### 2.5 Statistical analysis

One-way analysis of variance (ANOVA) analyzed the data, and Tukey’s *post hoc* test assessed the differences. SPSS version 16.0 (SPSS Inc., Chicago, Release 16.0.0, 2007) was used in all statistical calculations. Results were judged statistically significant at *p*-value <0.05. Values are averages and standard deviations for 3 independent replicates.

## 3 Results

### 3.1 Degree of hydrolysis and free amino acids

β-lactoglobulin (BLG) was hydrolyzed with trypsin at a 1:200 E/S ratio (enzyme: substrate) under the optimal conditions for a 120-min total period. The degree of hydrolysis (B) and the free amino acids concentration (C) were estimated in BLG hydrolysates (B) at different intervals, 30–120 min. Over time, the degree of enzymatic degradation gradually increased from 8.96 after 30 min to 26.01% after 120 min. The variations in DH are typically due to the enzymatic reaction time, influencing the breaking of peptide bonds. The results also showed a parallel increase in the free amino acid level as it increased from 2.51% at 30 min to 5.84% at 120 min.

### 3.2 Sodium dodecyl sulfate gel electrophoresis (SDS-PAGE)

**FIGURE 1:**
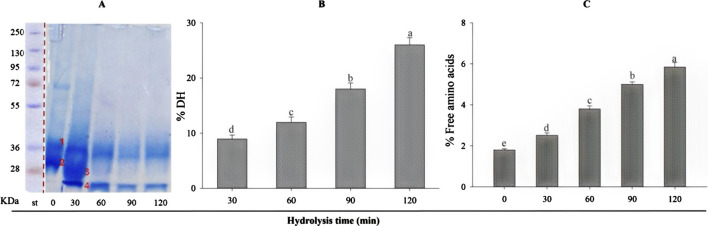
**(A)** SDS-PAGE of β-lactoglobulin. Purified BLG showed two bands at zero time (1 and 2), then two new bands appeared after 30 min (bands 3 and 4). The degree of tryptic hydrolysis (DH %) of β-lactoglobulin (E/S = 1:200) during 120 min at 37°C and pH 8 **(B)**, and the relative content (%) of free amino acid **(C)**. Values indicated by different small letters **–**(a, b, c, d, e) are significantly different according to Tukey’s HSD test (*p* ≤ 0.05).

SDS-PAGE qualitatively estimated the extent of β-lactoglobulin degradation by trypsin (Figure 1), exhibiting the electrophoretic patterns produced during the 120-min tryptic hydrolysis. It can be noticed that the purified BLG showed two bands at zero time (1 and 2), then two new bands appeared after 30 min (bands 3 and 4), the third is intermediate and the fourth is low-sized fragments. With increasing the hydrolysis time, the intermediate band (band 3) disappeared while the low-sized one (band 4) remained but faded gradually with time. So, it can be understood that in the first 30 min, the band fissure targeted the bonds in the middle of the molecule, while with time, the degradation targeted the distal parts of the peptide fragments, leading to the disappearance of the staining color. It is expected then that small-sized peptides will be predominant with increasing hydrolysis time. Also, band No 1, appearing in the native BLG resisted hydrolysis and was only partially degraded.

### 3.3 Fourier transform infrared (ftir) spectroscopy of β-lactoglobulin before and after hydrolysis

**FIGURE 2:**
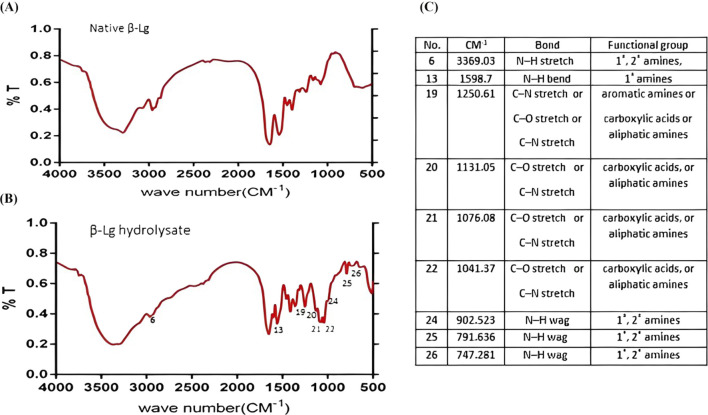
IR spectra of native β-lactoglobulin **(A)** and 1-h tryptic β-lactoglobulin hydrolysate (E/S = 1:200) at 37°C and pH 8 **(B)**. Characteristic IR absorption peaks of functional groups **(C)**. CM^-1^ is the wave number at which the specific chemical bond is absorbing the IR wave.

Changes in the chemical structure of BLG before and after hydrolysis with trypsin for different times were estimated using FTIR spectroscopy (Figure 2). Peaks for intact BLG (Figure 2) were found at 3,349, 2,917, 2,284, 1,657, 1,507, 1,390, 1,240, 1,056, 706, and 567 Cm^-1^. The FTIR spectra of BLG 60-min tryptic hydrolysate showed nine new peaks which were absent in that of the intact protein, i.e., at 3,369, 1,598, 1,250, 1,131, 1,076, 1,041, 902^,^ 791 and 747 Cm^-1^, referring probably to the free carboxylic, and amine groups released during the hydrolysis process. This confirms the hydrolysis process.

### 3.4 Nano-LC MS/MS analysis (LCMS)

**FIGURE 3:**
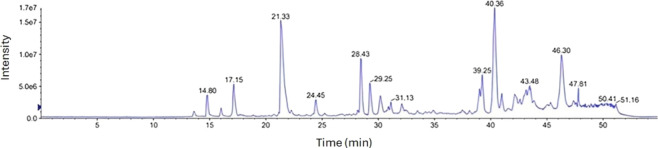
Mass spectrometric chromatogram (LCMS/MS) of peptides formation from tryptic β-lactoglobulin hydrolysate (E/S = 1:200) for 1 h at 37°C and pH 8 by (LCMS/MS).

LCMS estimated the peptide components of the BLG 1-h tryptic hydrolysate (37°C and pH8) at the positive ion mode are shown in (Figure 3; Tables 1, 2). The analysis produced initially 1,444 peaks which were translated into only 162 peptides of molecular masses ranging from 800 to 5671Da. The peptide population can be classified according to the molecular weight (MW) into 24 low-MW peptides (800–1,000 Da) and 37 high-MW (2000–3,000 Da) shown in Table 1 and 82 moderate-MW peptides (1,000–2000 Da) as shown in Table 2. Additionally, 19 extra-large-MW peptides (>3,000 Da) were obtained and included in the supplementary data (Supplementary file 1).

**TABLE 1:** LCMS detected low-MW (800–1,000) peptides (A) and High-MW (1,000–2,000) peptides (B) produced from 1.0-h tryptic hydrolysis of BLG (E/S = 1:200), under optimal conditions (37°C and pH 8).

(A) Low-MW peptides				
Serial	MW	Sequence	Freq	AA NO
1	800	KGLDIQK	3	7
2	804	LIVTQTM	3	7
3	804	LIVTQTMK	88	8
4	813	KIIAEK	3	6
5	819	IVTQTMK	3	7
6	823	KVAGTWY	2	7
7	828	GLDIQKV	2	7
8	828	GLDIQK	2	7
9	836	ALPMHIR*	55	7
10	838	DAQSAPLR*	2	8
11	851	VAGTWY	1	6
12	852	VLDTDYK	3	7
13	881	LDTDYKK	4	7
14	898	IDALNENK	24	8
15	902	TKIPAVFK	15	8
16	936	VLVLDTDY	1	8
17	936	VLVLDTDYK	1	9
18	949	ALPMHIRL*	3	8
19	964	KALPMHIR*	6	8
20	969	LDAQSAPLR*	1	9
21	970	DLEILLQK	1	8
22	973	EALEKFDK	2	8
23	980	VLDTDYKK	7	8
24	990	AMAASDISLL	2	10
B) High-MW peptides				
1	2001	TPEVDDEALEKFDKALK	27	17
2	2012	SLAMAASDISLLDAQSAPLR	60	20
3	2050	VEELKPTPEGDLEILLQK	25	18
4	2012	TKIPAVFKIDALNENKVL	1	18
5	2056	VYVEELKPTPEGDLEILL	5	18
6	2063	ALKALPMHIRLSFNPTQL*	14	18
7	2090	IDALNENKVLVLDTDYKK	20	18
8	2090	KIDALNENKVLVLDTDYK	2	18
9	2,192	YSLAMAASDISLLDAQSAPLR	1	21
10	2,168	EALEKFDKALKALPMHIR*	4	18
11	2,184	VYVEELKPTPEGDLEILLQ	2	19
12	2,218	KIDALNENKVLVLDTDYKK	18	19
13	2,243	LLLALALTCGAQALIVTQTMK*	5	21
14	2,264	LIVTQTMKGLDIQKVAGTWY	1	20
15	2,291	SLAMAASDISLLDAQSAPLRVY	8	22
16	2,312	VYVEELKPTPEGDLEILLQK	82	20
17	2,327	YVEELKPTPEGDLEILLQK	2	19
18	2,354	TKIPAVFKIDALNENKVLVL	1	20
19	2,354	IIAEKTKIPAVFKIDALNENK	7	21
20	2,381	KIDALNENKVLVLDTDYKKY	1	20
21	2,423	FKIDALNENKVLVLDTDYKK	10	20
22	2,452	CQCLVRTPEVDDEALEKFDK	2	20
23	2,468	RVYVEELKPTPEGDLEILLQK	11	21
24	2,476	ALPMHIRLSFNPTQLEEQCHI	3	21
25	2,482	KIIAEKTKIPAVFKIDALNENK	1	22
26	2,498	VYVEELKPTPEGDLEILLQKW	1	21
27	2,524	VFKIDALNENKVLVLDTDYKK	19	21
28	2,624	LRVYVEELKPTPEGDLEILLQK	2	22
29	2,660	TPEVDDEALEKFDKALKA	2	18
30	2,706	VAGTWYSLAMAASDISLLDAQSAPLR	34	26
31	2,723	AGTWYSLAMAASDISLLDAQSAPLR	7	25
32	2,739	GTWYSLAMAASDISLLDAQSAPLR	7	24
33	2,739	TWYSLAMAASDISLLDAQSAPLR	6	23
34	2,746	IPAVFKIDALNENKVLVLDTDYKK	1	24
35	2,861	TPEVDDEALEKFDKALKALPMHIR*	3	24
36	2,968	VAGTWYSLAMAASDISLLDAQSAPLRVY	5	28
37	2,975	TKIPAVFKIDALNENKVLVLDTDYKK	10	26

*High biological activity according to the Peptide Ranker site. Yellow (basic hydrophobic peptides). Blue (neutral hydrophobic peptide) Green (acidic hydrophobic peptides). These colors apply for Table 1 and 2.

**TABLE 2:** LCMS detected Medium-MW peptides produced from 1.0-h tryptic hydrolysis of BLG (E/S = 1:200), under optimal conditions (37°C and pH 8).

Serial	MW	Sequence	Freq	AA No
1	1,025	KIDALNENK	37	9
2	1,027	GDLEILLQK	1	9
3	1,032	TMKGLDIQK	1	9
4	1,045	ALIVTQTMK	1	9
5	1,046	EVDDEALEK	1	9
6	1,060	LIVTQTMK	5	8
7	1,063	WENGECAQK	5	9
8	1,064	VLVLDTDYK	18	9
9	1,077	SLAMAASDISL	1	11
10	1,093	DEALEKFDK	1	9
11	1,093	LVLDTDYKK	3	9
12	1,097	VAGTWYSLAM*	1	10
13	1,099	IPAVFKIDAL*	2	10
14	1,114	QALIVTQTMK	2	10
15	1,118	DAQSAPLRVY	3	10
16	1,126	IIAEKTKIPA	5	10
17	1,127	IDALNENKVL	3	10
18	1,130	TKIPAVFKID	2	10
19	1,148	ALKALPMHIR*	7	10
20	1,176	LSFNPTQLEE	1	10
21	1,190	SLAMAASDISLL	21	12
22	1,191	WENGECAQKK	7	10
23	1,192	VLVLDTDYKK	35	10
24	1,199	IPAVFKIDALN	1	11
25	1,226	TPEVDDEALEK	19	11
26	1,255	KIDALNENKVL	7	11
27	1,282	ISLLDAQSAPLR	1	12
28	1,304	LSFNPTQLEEQ	2	11
29	1,305	SLAMAASDISLLD	1	13
30	1,320	KVLVLDTDYKK	2	11
31	1,328	SDISLLDAQSAPL	3	13
32	1,328	IIAEKTKIPAVF	5	12
33	1,328	IIAEKTKIPAVFK	28	13
34	1,328	TKIPAVFKIDAL	14	12
35	1,349	GLDIQKVAGTWY	6	12
36	1,355	VLVLDTDYKKY	4	11
37	1,359	VYVEELKPTPEG	1	12
38	1,391	TPEVDDEALEKF	1	12
39	1,397	GLDIQKVAGTWY	6	12
40	1,397	DISLLDAQSAPLR	2	13
41	1,428	TKIPAVFKIDALN	2	13
42	1,428	EVDDEALEKFDK	2	12
43	1,436	KIIAEKTKIPAVF	3	13
44	1,456	KIIAEKTKIPAVFK	8	14
45	1,469	IDALNENKVLVL	1	12
46	1,470	AASDISLLDAQSAPL*	3	15
47	1,484	SDISLLDAQSAPLR	8	14
48	1,504	SLAMAASDISLLDAQ	3	15
49	1,506	TPEVDDEALEKFD	2	13
50	1,544	TPEVDDEALEKFDK	81	14
51	1,549	SFNPTQLEEQCHI	3	13
52	1,555	GLDIQKVAGTWYSL*	1	14
53	1,570	ASDISLLDAQSAPLR	3	15
54	1,586	IPAVFKIDALNENK	5	14
55	1,592	LIVTQTMKGLDIQK*	8	14
56	1,616	FDKALKALPMHIR	20	13
57	1,620	TPEVDDEALEKFDK*	81	14
58	1,626	GLDIQKVAGTWYSLA*	1	15
59	1,657	AASDISLLDAQSAPLR	3	16
60	1,672	LSFNPTQLEEQCHI	51	14
61	1,680	AMAASDISLLDAQSAPL	2	17
62	1746	VEELKPTPEGDLEIL	4	15
63	1750	SDISLLDAQSAPLRVY*	1	15
64	1751	DKALKALPMHIR*	2	12
65	1757	GLDIQKVAGTWYSLAM	1	16
66	1762	MAASDISLLDAQSAPLR	2	17
67	1790	TPEVDDEALEKFDKA	18	15
68	1793	VEELKPTPEGDLEILL	2	16
69	1800	TKIPAVFKIDALNENK	11	16
70	1822	ELKPTPEGDLEILLQK	1	16
71	1828	AMAASDISLLDAQSAPLR	3	18
72	1862	LALTCGAQALIVTQTMK	1	17
73	1867	VAGTWYSLAMAASDISLL	1	18
74	1872	SLAMAASDISLLDAQSAPL	16	19
75	1889	VRTPEVDDEALEKFDK	12	16
76	1893	GLDIQKVAGTWYSLAMAA*	1	18
77	1922	VEELKPTPEGDLEILLQ	2	17
78	1943	VYVEELKPTPEGDLEIL	3	17
79	1946	TPEVDDEALEKFDKALK	6	17
80	1962	IDALNENKVLVLDTDYK	2	17
81	1969	IRLSFNPTQLEEQCHI	2	16
82	1997	SLAMAASDISLLDAQSAPLR	2	20

It can be generally remarked that most of the peptides were of hydrophobic nature while combined with either basic nature or acid nature. In the low-MW peptides, there were nine hydrophobic basic (HB) representing 38% while hydrophobic acidic (HA) peptides were only seven representing 29%. These HB peptides may result in considerable biological activity of the hydrolysate. As for the high-MW (2000–3,000) peptides there were only 9 HB peptides representing only 24% of the total against 59% for the HA peptides and in the moderate-MW there were 20 HB representing 24% while HA peptides were 44 representing 53%.

In this study an initial analysis of peptides was conducted using Electro-spray-ionization-mass spectrometry (ESI-MS) estimated the peptide components of the BLG tryptic hydrolysates at the positive ion mode giving less information and reliability. However, there were certain similarities in the overall trend of results. The main peaks resulting from trypsin (Supplementary file 2) included 81 peptides of molecular masses ranging from 164.83 to 903.52 Da. These comprised 37 dipeptides, seven tripeptides, 14 tetrapeptides, 11 pentapeptides, 5 hexapeptides and 3 heptapeptides. Quantitatively, the dipeptides represented 15.5% of the total peptides, despite their relatively high number (37). The rest of the peptides represented about 84.5% of the total and consisted of tripeptides (17.8%), tetrapeptides (31.6%), pentapeptides (10.9%), hexapeptides (19.5) and heptapeptides (4.5%). Thus, tetrapeptides quantitatively represented the major components, followed by hexapeptides and tripeptides. A comparison between these two methods of identifying the liberated peptides is shown in the Supplementary file, Table 5s.

### 3.5 Antioxidant activity

#### 3.5.1 DPPH radical scavenging activity

**FIGURE 4:**
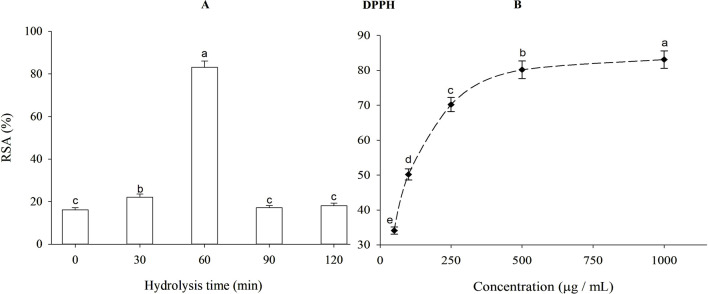
DPPH free radical scavenging activity (RSA) of β-lactoglobulin (BLG) hydrolysates) 500 μg/mL) produced by trypsin (E/S = 1:200) at 37°C and pH 8 at different time intervals **(A)**, and DPPH radical scavenging activity of 1-h tryptic BLG hydrolysates (the highest antioxidant activity) at different concentrations **(B)**. Different small letters (a, b, c, d, e) indicate significant differences following Tukey’s HSD test (*p* ≤ 0.05).

Antioxidants are well-known for their ability to transform free radicals into stable species, stopping the oxidation process. As a free radical, DPPH has been extensively used to evaluate reducing substances. DPPH radical scavenging activities of β-lactoglobulin hydrolysates produced using trypsin at different times are depicted in Figure 4A. Increasing the degree of hydrolysis of β-lactoglobulin from 8.96% to 11.97%, significantly (*p* < 0.05) and highly increased the DPPH radical scavenging activity from 16.17% to 83.11%, i.e., after 1-h hydrolysis. This antioxidant capacity is very close to that produced by TBHQ, i.e., 89.17% (data do not show).

The DPPH radical scavenging activity produced by 60-min tryptic hydrolysate (the highest antioxidant activity) at different concentrations is depicted in Figure 4B. When the concentration of 1-h tryptic β-lactoglobulin hydrolysate was increased from 50 to 1,000 μg/mL, the DPPH radical scavenging activity augmented significantly (*p* < 0.05) from 34.13% to 83.11%.

#### 3.5.2 Ferric reducing antioxidant power (FRAP)

**FIGURE 5:**
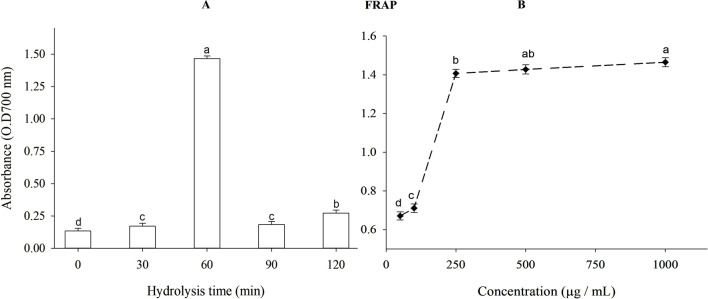
**(A)** Ferric reducing antioxidant power (FRAP) of β-lactoglobulin (BLG) hydrolysates produced by trypsin (E/S = 1:200) at 37°C and pH 8 at different time intervals and **(B)** FRAP of BLG hydrolysates with the highest antioxidant activity (produced after 60 min tryptic hydrolysis) at different concentrations. Different small letters refer to significant differences according to Tukey’s HSD test (*p* ≤ 0.05).

The ferric reducing antioxidant power (FRAP) of β-lactoglobulin (BLG) hydrolysates produced using trypsin at different times is shown in Figure 5A. The intact protein exhibited a weaker reducing action than the hydrolysates (*p* < 0.05). After 60 min, hydrolyzed BLG had a higher FRAP value (absorbance = 1.465) and DH = 11.97% compared to 1.476 for TBHQ (data not shown). The FRAP of BLG 60-min tryptic hydrolysates at different concentrations is shown in Figure 5B. When the concentration of β-lactoglobulin hydrolysates was increased from 50 to 1,000 mg/mL, the FRAP value rose significantly (*p* < 0.05) from 0.671 to 1.465.

#### 3.5.3 β-Carotene bleaching method

Since β-carotene is exceptionally susceptible to linoleic acid-free radicals-mediated oxidation, this assay is appropriately utilized to evaluate the antioxidant activity of bioactive compounds. Therefore, the discoloration of β-carotene by linoleic acid oxidation was determined under the influence of intact BLG or its tryptic hydrolysate at diversified time intervals.

**FIGURE 6:**
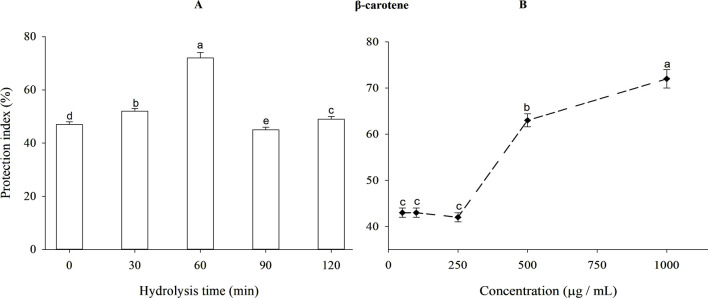
Antioxidant protective index (%) of β-lactoglobulin (BLG) hydrolysates produced using trypsin hydrolysis at different times (0–120 min), E/S = 1:200, at 37°C and pH 8 **(A)** and the antioxidant protective index (%) of 60-min tryptic β-lactoglobulin (BLG) hydrolysate showing the highest antioxidant activity at different concentrations **(B)**. Different lowercase letters refer to significant differences among the hydrolysis times **(A)** and concentrations **(B)** according to Tukey’s HSD test (*p* ≤ 0.05). The protection index (PI) was calculated following the equation: Protection index (%) = A/A0 × 100, wherein A0 sample is the absorbance of the sample at zero-time, A is the absorbance after 120 min.

Antioxidant activity after 120 min of reaction for BLG hydrolysates produced using trypsin at different times, expressed as protection index is presented in Figure 6A. Unhydrolyzed BLG presented 47% protection index. The antioxidant action increased with time during the initial stages of hydrolysis, reaching its maximum peak after 60 min of trypsin hydrolysis. The antioxidant effect diminished as the DH increased after this point. After 60 min, hydrolyzed BLG had a higher protection index (72%) than the value (59.3%) achieved by TBHQ (data do not show). The protection index of 60-min BLG tryptic hydrolysate at different concentrations appears in Figure 6B. The protection index increased significantly (*p* < 0.05) from 42% to 72% as concentrations increased from 250 to 1,000 μg/mL.

### 3.6 Anticancer activity

#### 3.6.1 Cell viability *in vitro*

**FIGURE 7:**
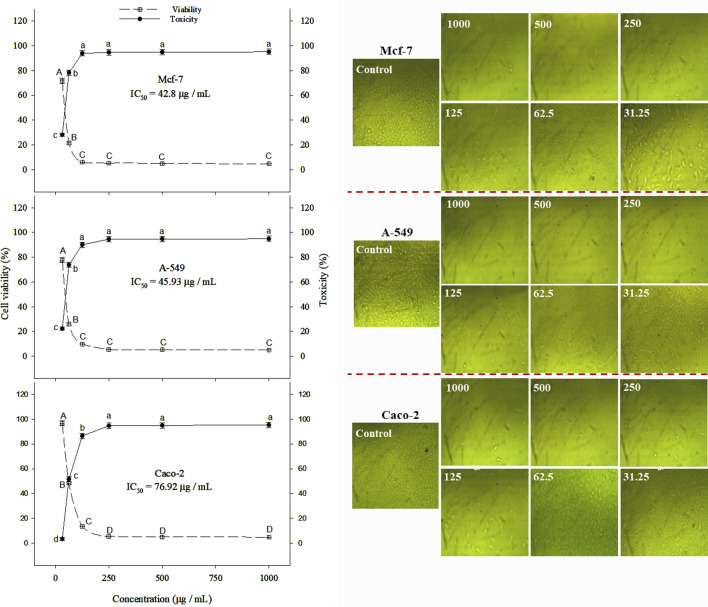
Toxicity (%) and cell viability (%) of Mcf-7, Caco-2, and A-549 cancer cell lines treated with BLG hydrolysates produced using trypsin (E/S = 1:200) for 60 min at 37°C and pH 8 (the highest antioxidant activity) at different concentrations. Different letter indicates significant differences among the cell viability (capital letters) and toxicity (small letters) according to Tukey’s HSD test (*p* ≤ 0.05). IC_50_ is the hydrolysate concentration producing 50% growth inhibition.

**FIGURE 8:**
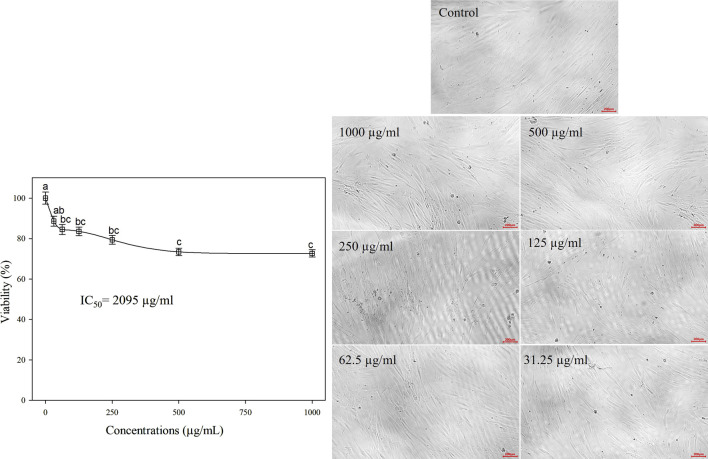
Cell viability (%) of FSU normal cell line treated with BLG hydrolysates produced using trypsin (E/S = 1:200) for 60 min at 37°C and pH 8 (the highest antioxidant activity) at different concentrations. Different letter indicates significant differences among the cell viability according to Tukey’s HSD test (*p* ≤ 0.05). IC_50_ is the hydrolysate concentration producing 50% growth inhibition.

β-lactoglobulin 60-min tryptic hydrolysate (producing the highest antioxidant activity) was tested *in vitro* for its anticancer activity against Mcf-7, Caco-2, and A-549 human cancer cell lines at different concentrations in the range 31.25–1,000 µg/mL, using the MTT assay. The linear relationship between cell viability (%) and BLG hydrolysate concentration and the overall cell viability (%) decreased with increasing the concentration of BLG hydrolysates. The MTT assay revealed an inhibitory action of β-lactoglobulin hydrolysates on the proliferation of the human cancer cell lines (Mcf-7, Caco-2, and A-549) in a concentration-based manner (Figure 7). β-Lactoglobulin hydrolysates exhibited the lowest IC_50_ against Mcf-7 at 42.8 μg/mL, while the highest value 76.92 μg/mL was realized against Caco-2. It can be concluded that β-lactoglobulin 60-min tryptic hydrolysates is generally effective against the three studied carcinogenic cell lines, where IC_50_ was in the range 42.8–76.92 μg/mL, particularly effective against Mcf-7 cell line. The cytotoxicity of BLG 60-min tryptic hydrolysate at concentrations ranging from 31.25 to 1,000 μg/mL was evaluated in FSU cells, with results shown in Figure 8. The IC50 of β-lactoglobulin hydrolysate was recorded at 2095 μg/mL.

#### 3.6.2 Quantification of mRNA levels of Caspase-9

**FIGURE 9:**
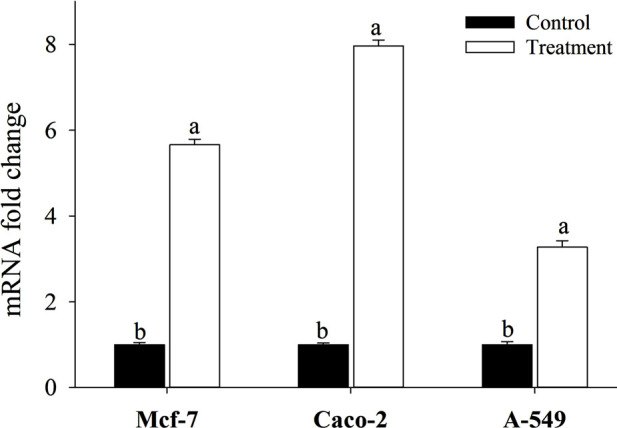
Effect of 60-min tryptic BLG hydrolysate (E/S = 1:200) at 37°C and pH 8 (the highest antioxidant activity) on caspase 9 mRNA expression of three human cancer cell lines (Mcf-7, Caco-2, and A-549). Cells were treated with the concentration causing the IC_50_ for each cell line for 24 h and their mRNA levels were evaluated by quantitative real-time PCR. Different small letters (a, b) refer to significant differences according to Tukey’s HSD test (*p* ≤ 0.05). IC_50_ is the hydrolysate concentration producing 50% growth inhibition.

Investigating the molecular mechanism of apoptosis in human cancer cell lines (Mcf-7, Caco-2, and A-549) induced by the 60-min tryptic BLG hydrolysate necessitated estimating caspase-9 expression (Figure 9). Gene expression of Caspase-9 transcripts seems highly multiplied by the application of BLG hydrolysates. The expression of caspase-9 in Mcf-7, Caco-2, and A-549 cells treated with 42.8, 76.92, and 45.93 μg/mL BLG hydrolysates for 24 h was multiplied by 5.659, 7.965, and 3.275-fold, respectively, as compared to the levels of the untreated control cells.

#### 3.6.3 *In vitro* VEGFR-2 kinase

**FIGURE 10:**
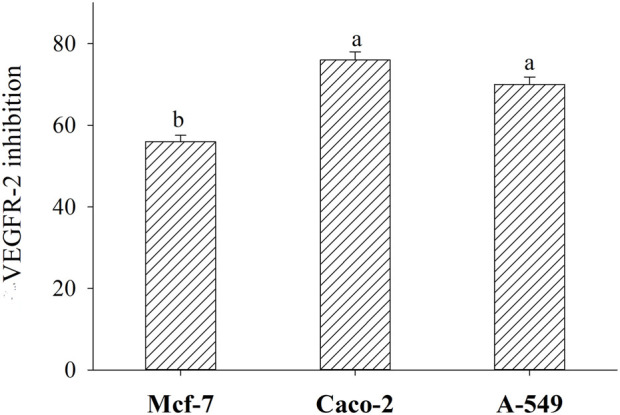
Inhibition (%) of Vascular endothelial growth factor receptor (VEGFR-2) in three human cancer cell lines (Mcf-7, Caco-2, and A-549) after treatment with 60-min tryptic BLG hydrolysate (E/S = 1:200) at 37°C and pH8 (IC50 for each cell line). Different small letters refer to significant differences according to Tukey’s HSD test (*p* ≤ 0.05). Anticarcinogenic Cationic Peptide.

In the current study, BLG 60-min tryptic hydrolysate with the most antiproliferative action against Mcf-7, Caco-2, and A-549, with IC50 amounting, respectively, to 42.8, 76.92, and 45.93 μg/mL, were evaluated for their inhibitory activities against VEGFR-2, using an anti-phosphotyrosine antibody within PerkinElmer Alpha Screen system (Figure 10). It is observed that BLG 60-min tryptic hydrolysate strongly inhibited VEGFR-2, the most significant transducer of the vascular endothelial growth factor (VEGF)-dependent angiogenesis, in Mcf-7, Caco2, and MA-549 cells by about 56%, 76%, and 70%, respectively.

## 4 Discussion

This study aimed to investigate the antioxidant and anticancer effects of the hydrolysis of BLG. The data indicated apparent anticancer effects via affecting proliferation, increasing apoptosis, and inhibiting angiogenesis. This anticancer action is principally due to the hydrolysis of BLG into active basic and hydrophobic natural peptides with radical scavenging activity. Hydrophobic peptides can easily interact with the lipid bilayers of cancer cell membranes, enabling their disruption and leading to cell lysis and death (El-Didamony et al., 2024). The hydrophobic regions of these peptides allow them to insert into the cell membrane, creating pores or channels that compromise the cell’s structural integrity (Huang et al., 2011). Likewise, the basic Character of peptides allows them to bind to the negatively charged components of cancer cell membranes, e.g., as phospholipids and glycoproteins, allowing them to penetrate the cell membrane and exert their cytotoxic effects inside the cells (El-Didamony et al., 2024). Moreover, the positively charged peptides can also interact with intracellular targets, further promoting anticancer activity (Huang et al., 2011).

The increasing degree of hydrolysis of BLG over the time course, reaching 26.01% after 120 min, is typically due to the enzymatic reaction time, influencing the breaking of peptide bonds (Abdel-Hamid et al., 2017). The results also showed a parallel increase in free amino acid levels, which indicates that a considerable portion of the protein molecules was fragmented into free amino acids. The degree of hydrolysis after 1 h, 11.97% may seem ideal and amounts to about 46% of the maximum degree of hydrolysis achieved after 2 hours. This hydrolysis degree was later realized as the one associated with the highest antioxidant activity and thus selected for further analysis and biological application.

The SDS-PAGE separation indicated the appearance of two new bands after 30 min, one intermediate and the other a low-sized fragment. With increasing the hydrolysis time, the intermediate band (band 3) disappeared while the low-sized only faded gradually with time. So, it can be understood that in the first 30 min, the band fissure targeted the bonds in the middle of the molecule, while with time, the degradation targeted the distal parts of the peptide fragments, leading to the disappearance of the intermediate peptides. The electrophoretic patterns after 1–2 h remained nearly the same and were characterized by the dominance of low molecular weight peptides. Similar results were shown previously (Garcia-Mora et al., 2015; Garcia-Mora et al., 2016). The FTIR spectra of BLG 60-min tryptic hydrolysate showing nine new peaks, which were absent in that of the intact protein, refer probably to the free carboxylic, and amine groups released during the hydrolysis process and confirm the occurrence of the enzymatic degradation.

The LCMS of BLG 60-min tryptic hydrolysate exhibited the release of 162 peptides of molecular masses ranging from 800 to 5,671 Da, which were classified according the molecular weight (MW) into 24 low-MW peptides (800–1,000 Da), 37 high-MW (2000–3000Da), 82 moderate-MW peptides (1,000–2000) and 19 extra-large-MW peptides (>3,000 Da). It was recognized that the highest number of peptides was within the moderate-MW. Alternatively, most of the peptides were of hydrophobic nature combined with either basic nature or acidic character. In the low-MW there were nine hydrophobic basic (HB) representing 38% while hydrophobic acidic (HA) peptides were only seven representing 29%. This HB peptides may result in considerable biological activity of the hydrolysate. This composition, characterized by the dominance of basic and hydrophobic peptides, may have contributed to the anticarcinogenic activity.

Likewise, most of the higher-sized peptides, high-MW (1,000–2000Da), also showed hydrophobic basic characters and representing 24%. The high level of the HB peptides may be because trypsin action is known to favor the hydrophobic nature of the protein substrate and specifically target the basic amino acid residues. The relatively higher proportions of the HB peptides in the 1-h BLG tryptic hydrolysate may indicate their higher potential as antioxidants and anticancer agents, may explain the relatively higher biological activity of this 1-h BLG-tryptic hydrolysate. Further hydrolysis may degrade these peptides to smaller sizes with potentially lower biological activities. However, further studies may be needed to discern which of these peptide classes or individual peptides may have the highest biological and anticancer activities.

The observed high increase in DPPH radical scavenging activity of BLG hydrolysate from 16.17% (after 30 min) to 83.11%, (after 60 min) hydrolysis coincided with an increase in the degree of hydrolysis from 8.96% to 11.97%, indicate that releasing the free amino and carboxyl groups have an enormous effect on the scavenging capacity of the product, that it approached the value of TBHQ, (89.17%). The significant increase in DPPH radical scavenging activity of 60 min tryptic BLG hydrolysate when increasing the concentration from 50 to 1,000 μg/mL confirms the high antioxidant capacity of this product. According to this finding, the BLG hydrolysate might have been an effective electron donor and could combine with free radicals to end the radical chain reaction. This action might arise from the components rich in electron density as the released free amino and carboxyl groups. The amount of histidine and other hydrophobic amino acids in β-lactoglobulin and their concentrations are related to its antioxidant activity (Grażyna et al., 2017). Thus, through the free radical scavenging activity, the BLG tryptic hydrolysate can protect the cells against excessive oxygen stress in accordance with (Galano et al., 2013). With hydrolysis, BLG showed also higher ferric reducing antioxidant power (FRAP). After 60 min, hydrolyzed BLG hydrolysate had a value very close to TBHQ, confirming its potent antioxidant activity. This activity can be also enhanced when increasing the concentration of this product.

Tryptic BLG hydrolysate could also protect β-carotene against linoleic acid-free radicals-mediated oxidation. This protective action increased with increasing the DH reaching its maximum after 1 hour (72%) which excelled the action of TBHQ (59.3%), confirming the strength of this product. The protection index of 60-min BLG tryptic hydrolysate was concentration-dependent. Inhibiting lipid peroxidation may be a result of the strong emulsifying ability of the hydrolysate based on its high content of hydrophobic amino acids, which can expose more active sites of the peptide, to be accessible to the chain reaction of lipid oxidation (Hirose and Miyashita, 1999).

Peptides have strong antioxidant capacity, particularly when they contain considerable hydrophobic amino acid residues. Typically, structural antioxidant peptides include hydrophobic amino acid residues, e.g., Leu or Val at the N-terminus and Pro, His, or Tyr in the middle (Sabeena Farvin et al., 2010). According to Zou et al. (2016), Trp, Phe, Val, Ile, Gly, Lys, and Pro are the most known hydrophobic amino acids involved with antioxidant activities. Tian et al. (2015) concluded that the hydrophobic Trp residues contribute to the high antioxidant activity of peptides. Likewise, Pro residue may alter the secondary structure of peptides, thus promoting the interaction of the peptide amino acid residues and their antioxidative properties (Zou et al., 2016). Additionally, Leu and the motifs Ser-Leu, Thr-Leu, and Pro-Leu, mainly located at the N- and C- terminals, enhance the antioxidant activities of peptides, while the C-terminus is more influential in determining the activity.

Li and Li (2013). Moreover, the aromatic rings of hydrophobic Phe and Tyr residues can interact with hydroxyl radicals, producing stable derivatives (Pownall et al., 2010). Alternatively, hydrophobic residues are suggested to promote the interaction of antioxidant peptides with the hydrophilic targets of oxidation. A positive relationship was established between the total content of hydrophobic amino acids of food protein hydrolysates and their capacity to scavenge a hydrophobic synthetic radical (Udenigwe and Aluko, 2011).

So, the diversity and multiplicity of the peptides in the hydrolysate may stand behind this distinct high capacity. Since the 60-min tryptic hydrolysate was particularly associated with the highest activity, it can be concluded that the antioxidant activity is highly peptide size-dependent as further hydrolysis deteriorated this activity in accordance with (Wen et al., 2020). In a recent study, six novel peptides with a molecular weight of the range 385–785 exhibited strong antioxidant potential against *in vitro* radicals (Tyagi et al., 2023). Collectively, it can be stated that the antioxidant action of the released peptides is due to their ability to scavenge free radicals based on their structural and size specifications as well as their amino acid composition and sequence following (Vibhute et al., 2023). The antioxidant activity observed here is essentially based on the capacity of the peptides to scavenge free radicles, agreeing with Lv et al. (2022). It has been reported that the protease specificity, degree of hydrolysis, and nature of the released peptides play significant roles in the capacity of the antioxidant activity of proteins following hydrolysis (Tang et al., 2009; Yust et al., 2012).

β-lactoglobulin hydrolysate was shown to have inhibitory effect on proliferation of different kinds of human cancer cell lines (Mcf-7, Caco-2, and A-549), using MTT assay, in a concentration-based manner proves general effectiveness of this product particularly when IC_50_ was in the range 42.8–76.92 μg/mL against the three tested cell-lines, where Mcf-7 showed the least value. At the same time, β-lactoglobulin hydrolysates appeared to promote apoptosis by increasing the expression levels of Caspases nine by about 3-8 folds in human cancer cells. The ability of many anticancer drugs to induce apoptosis may construct their mechanism of action (Sen and D'Incalci, 1992; Motomura et al., 2008).

The peptides’ amino acid composition can potentially exert anticancer activity against several types of cancer cells directly. As an illustration, peptides with a high cationic can potentially augment their specific activity toward cancer cells. However, an elevation in hydrophobic peptides may lead to a reduction in the level of specificity (Chiangjong et al., 2020). Caspase-9 is a key player in various stimuli, including chemotherapies, stress agents, and radiation. Caspase-9 should be activated via plenty of intrinsic proteins and small molecules to maintain its catalytic status; otherwise, pathophysiological outcomes may occur, leading to degenerative disorders or cancer (Li et al., 2017). Peptides rich in lysine and arginine, which possess an intact amphipathic helical interface, can augment cell lysis through membrane-lysis mechanisms. These peptides can breach the cell membrane and initiate caspase 3-dependent apoptotic cell death (Gabernet et al., 2019). Similarity, the anticancer activity of hydrophobic peptides was also attributed to membrane-lysis mechanism based on their fast necrotic action (Huang et al., 2011). Hence, using the tryptic BLG hydrolysate in this study has exerted its stimulative action on caspase-9 in a way like the intrinsic proteins and small molecules. Increasing apoptosis in cells can be accomplished by a variety of means. A meaningful mechanistic approach to cancer chemoprevention and chemotherapy may involve substances that increase apoptosis, a process that suppresses the proliferation of malignant cells (Alshatwi, 2010). However, many newly developed anticancer medications suffer from undesirable side effects and are resistant to treatment (Khan and Mlungwana, 1999). As a result, a considerable interest in developing safe and more effective anticancer agents based on natural compounds is steadily growing (Panchal, 1998). Proteins have been scientifically proven to be promoters in preventing certain diseases, including cancer, and are thought to be an essential source of therapeutic peptides (Ramkisson et al., 2020). The anticancer properties of food-derived protein hydrolysates and peptides may exert their actions by several well-defined and variable mechanisms, encompassing apoptosis, cell cycle arrest, intracellular signaling systems’ inhibition, immune system regulation, protease inhibition, and nucleic acid impairment (RCK Rajendran et al., 2017). Bioactive peptides and protein hydrolysates may exert their anticancer activities by orchestrating cellular DNA damage simulating the common bean-derived peptide GLTSK, whose DNA impairment function was ascribed to the overexpression of the histone γH2AX in HCT116 human colorectal cancer cells (Luna-Vital et al., 2016). Alternatively, the antiproliferative action of the tryptic hydrolysate agrees with the finding of (Chalamaiah et al., 2015), indicating that a polycationic selectively induced cell death in leukemia Jurkat T cells through a Caspase-3 -independent mechanism by accelerating mitochondrial depolarization.

The potent inhibition of VEGFR-2 in Mcf-7, Caco2, and MA-549 cells by applying the tryptic BLG hydrolysate is a very important result since VEGFR-2 is the most significant transducer of the vascular endothelial growth factor (VEGF)-dependent angiogenesis (Holmes et al., 2007). Consequently, the studied BLG 60-min tryptic hydrolysate can be considered an efficient anticancer factor. The VEGF signaling pathway plays a fundamental role in regulating tumor angiogenesis. The therapeutic potential of VEGF has been demonstrated in many human cancers (Niu and Chen, 2010). VEGFR-2 (Vascular endothelial growth factor receptor 2) is regarded as the most significant transducer of VEGF-dependent angiogenesis, being a primary target of angiogenesis-relevant kinases (Holmes et al., 2007). As a result, it is thought that inhibiting the VEGF/VEGFR signaling pathway is a promising therapeutic target for controlling tumor angiogenesis and preventing potentially subsequent tumor growth (Vayner and Ball, 2000; Tugues et al., 2011; Eldehna et al., 2015; Abou-Seri et al., 2016). Consequently, Aflibercept (VEGF Trap), a human soluble decoy receptor protein, has a high affinity for all the isoforms of VEGF-A, VEGF-B, and placental growth factor, inhibiting effectively early stages of angiogenesis and arteriovenogenes (Sitohy et al., 2017).

The anticarcinogenic action of the tryptic BLG hydrolysate may originate from their cationic nature following (Singh et al., 2023) who stated that the tryptic cationic peptides obtained from the hydrolysis of catfish muscle possessed anticancer activity or from the high antioxidant activity of these peptides in agreement with (Cruz-Gregorio et al., 2022), who stated that the coadministration of an immunomodulatory peptide and appropriate redox therapy could have a synergistic effect against malignancies. The association between antioxidant activity was also reported in two β-casein-derived peptides, which showed high scavenging activity against free radicals and anti-proliferative action against HT-29 colon cancer cell line (Sah et al., 2016).

The natural anticarcinogenic BLG cationic peptides obtained in this study offer a versatile and potent approach to cancer therapy, with a high potential to improve treatment outcomes and reduce side effects. These peptides can target cancer cells through mechanisms different from those of traditional drugs, potentially overcoming drug resistance and they can modulate the immune response, enhancing the body’s natural ability to fight cancer. Finally these bioactive peptides can be used in combination with other treatments to enhance overall efficacy and reduce the likelihood of resistance development.

## 5 Conclusion

The results of this study indicated direct anticancer and antiangiogenic actions of BLG hydrolysates on human cancer cells, where the 60-min BLG tryptic hydrolysate with the majority of low-MW peptides (24 peptides), in the range of 800–1,000 Da, having the highest biological activity, in consistence with their strong antioxidant activities (DPPH radical scavenging activity, Ferric reducing antioxidant power, and β-Carotene bleaching power). These biological and anticancer activities are based on the chemical constitution and nature of the BLG tryptic hydrolysate, which possesses high contents of HB peptides 38%, promoting its anti-carcinogenic activity. The tryptic hydrolysates are generally effective against the three studied carcinogenic cell lines (Mcf-7, Caco-2, and A-549, where IC50 were 42.8, 76.92, and 45.93 μg/mL) by affecting their proliferation as shown by the MTT assay, angiogenesis, by exerting inhibitory activities against VEGFR-2, which was strongly inhibited by about 56%, 76%, and 70%, respectively, and promoting apoptosis by increasing the expression levels of Caspases nine by about 3-8 folds in human cancer cells. Subsequent future studies may focus on maximizing the anticarcinogenic effectiveness of the peptides while isolating and evaluating the most prominent individual peptide and through variating the treatment conditions.

## Data Availability

The original contributions presented in the study are included in the article/Supplementary Material, further inquiries can be directed to the corresponding author.
