# Correction: Anticarcinogenic cationic peptides derived from tryptic hydrolysis of β-lactoglobulin

**DOI:** 10.3389/fmolb.2025.1660539

**Published:** 2025-08-18

**Authors:** Eman Ibrahem, Ali Osman, Hefnawy Taha, Mohamed F. Abo El-Maati, Basel Sitohy, Mahmoud Sitohy

**Affiliations:** ^1^ Department of Biochemistry, Faculty of Agriculture, Zagazig University, Zagazig, Egypt; ^2^ Department of Clinical Microbiology, Infection and Immunology, Umeå University, Umeå, Sweden; ^3^ Department of Diagnostics and Intervention, Oncology, Umeå University, Umeå, Sweden

**Keywords:** β-lactoglobulin, trypsin, antioxidant activity, anticancer activity, caspase, VEGFR-2

Supplementary Figure 2 was omitted from Supplementary Data Sheet 1. The file has now been replaced.

The original article has been updated.

