# Supplementary material for: Anticarcinogenic cationic peptides derived from tryptic hydrolysis of β-lactoglobulin
Source: Front Mol Biosci. 2025 Jan 7;11:1444457. doi: 10.3389/fmolb.2024.1444457 (PMC11757936; doi:10.3389/fmolb.2024.1444457)
Supplement: Supplementary file 1 [file DataSheet1.docx]

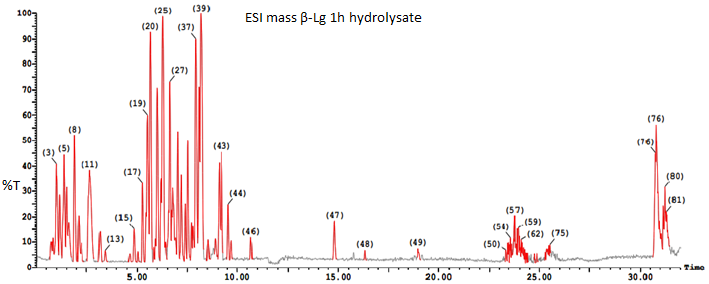


Supplementary Figure 1. Mass spectrometric chromatogram of peptides formation from tryptic β-lactoglobulin hydrolysate (E/S= 1:200) for 1h at 37 °C and pH 8 by electro‐spray‐ionization‐MS (ESI‐MS).


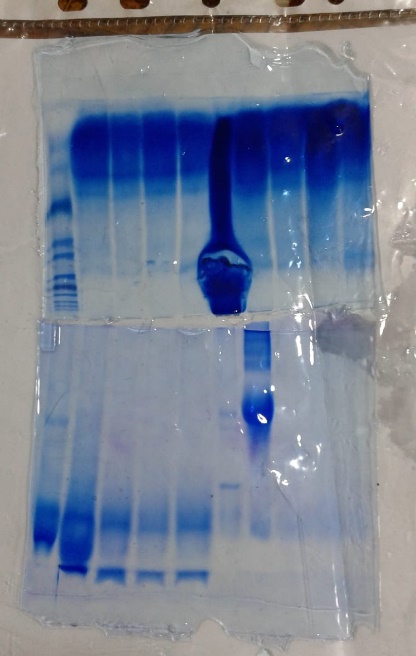


Supplementary Figure 2. The original image of SDS-PAGE of β-lactoglobulin named as Figure 1 in the published article. The original gel of the image appears in the lower half of this image (the second gel). The samples appearing in the article are the ones in the lanes 1-5 from the left.

Table 1s. Possible peptide compositions of 1.0-h tryptic BLG hydrolysate (E/S= 1:200),

produced at 37 °C and pH 8.

| **Peak No.** | **Total area %** | **Molecular weight (Da)** | **Composition** | |
| --- | --- | --- | --- | --- |
| 1 | 0.33 | 164.83 | PA | |
| 2 | 0.32 | 331.14 | CR | |
| 4 | 1.32 | 216.97 | DT | |
| 6 | 1.22 | 260.99 | MK | |
| 7 | 0.94 | 335.19 | CC | |
| 9 | 1.15 | 305.05 | CM | |
| 10 | 0.38 | 319 | YR | |
| 12 | 0.88 | 349 | CC | |
| 13 | 0.22 | 363 | WC | |
| 14 | 0.18 | 160 | SA | |
| 18 | 0.51 | 327 | YY | |
| 19 | 3.51 | 327.2 | YY | |
| 47 | 0.94 | 219 | MA | |
| 48 | 0.14 | 244 | EN | |
| 49 | 0.20 | 301 | WN | |
| 51:65 | 2.5 | 271.17 | RN | |
| 66-67 | 0.07 | 337.13 | CY | |
| 71-75 | 0.07 | 297.18 | CH | |
| Total | 15.37 |  |  | |
| Amino acids names followed the one-letter abbreviations: (https://www.genscript.com/Amino_Acid_Code.html) | | | |  |

**Table 2s. Classification of the di-peptides according to their contents of basic, acidic, and**

**hydrophobic amino acid residues**

| **Basic** | **Hydrophobic** | **Neutral** | **Acidic** |
| --- | --- | --- | --- |
| C**R**  MK  YR  RN  CH | PA  MK  WC  MA  WN | CC  CM  CC  AS  YY  YY  CY | **D**T  **E**N |
| **31.88 %** | **18.93299 %** | **47.625%** | **9.707%** |
| Basic: Red, Hydrophobic: Green, Acidic: Orange, Neutral: Black. The percentage was calculated relative to the total di-peptides. | | | |

Table 3s . Possible tri, tetra, Penta, hexa, and hepta peptide compositions of 1.0-h tryptic BLG hydrolysate (E/S= 1:200), produced at 37 °C and pH 8.

| Peak No. | Molecular weight (Da) | Peptide sequence | **Fragment**  **Sequence*** | Total area (%) | Relative proportion (%) |
| --- | --- | --- | --- | --- | --- |
|  |  | Dipeptides |  |  | 15.5 |
|  |  |  | Table (1) |  |  |
|  |  | Tripeptides |  |  | 17.79 |
| 15 | 393.0711 | FDK | 152-154 | 0.72 |  |
| 16 | 407.0563 | DYK or HIR | 114-116 or 162-164 | 0.18 |  |
| 31 | 388.2657 | QKK | 84-86 | 2.82 |  |
| 33 | 392.3021 | FDK | 152-154 | 1.04 |  |
| 76 | 413.2016 | YKK | 115-117 | 6.16 |  |
| 77 | 413.1741 | YKK | 115-117 | 0.56 |  |
| 76 | 413.2016 | Undefined |  | 6.16 |  |
| 77 | 413.1741 | Undefined |  | 0.56 |  |
|  |  | Tetrapeptides |  |  | 31.61 |
| 5 | 522.8799 | Undefined |  | 2.7 |  |
| 8 | 458.7635 | Undefined |  | 3.32 |  |
| 11 | 475.3417 | LPMH | 159-162 | 5.03 |  |
| 20 | 467.3981 | Undefined |  | 5.69 |  |
| 22 | 500.6954 | EELK | 60-63 | 0.28 |  |
| 25 | 546.1433 | Undefined |  | 7.86 |  |
| 26 | 534.4043 | DYKK | 114-117 | 0.24 |  |
| 34 | 546.315 | Undefined |  | 2.34 |  |
| 43 | 471.9813 | CLVR | 137-140 | 1.74 |  |
| 44 | 427.9628 | CAQK | 82-85 | 1.16 |  |
| 45 | 427.9698 | CAQK | 82-85 | 0.32 |  |
| 68 | 463.3936 | Undefined |  | 0.07 |  |
| 69 | 485.2285 | NENK | 104-107 | 0.04 |  |
| 70 | 463.3034 | Undefined |  | 0.08 |  |
|  |  | Pentapeptides |  |  | 10.91 |
| 3 | 573.334 | Undefined |  | 2.53 |  |
| 17 | 623.4188 | DTDYK | 112-116 | 1.6 |  |
| 21 | 561.2516 | Undefined |  | 0.16 |  |
| 24 | 654.1433 | EKFDK | 150- 154 | 1.3 |  |
| 28 | 638.2006 | TDYKK | 113-117 | 0.36 |  |
| 30 | 568.953 | EALEK | 147-151 | 0.3 |  |
| 32 | 578.2511 | Undefined |  | 1.93 |  |
| 36 | 618.3181 | DTDYK | 112-116 | 0.48 |  |
| 40 | 604.4654 | QCLVR | 136-140 | 0.38 |  |
| 42 | 555.2984 | DKALK | 153-157 | 1.71 |  |
| 50 | 678.6442 | Undefined |  | 0.16 |  |
|  |  | Hexapeptides |  |  | 19.5 |
| 27 | 696.4315 | Undefined |  | 3.76 |  |
| 29 | 681.0635 | KIIAEK | 86-91 | 1.4 |  |
| 37 | 684.5414 | KIIAEK | 146-151 | 5.64 |  |
| 39 | 772.1009 | Undefined |  | 8.17 |  |
| 41 | 744.1208 | LPMHIR | 159-164 | 0.37 |  |
|  |  | Heptapeptides |  |  | 4.49 |
| 35 | 838.2233 | LEILLQK | 70-76 | 0.58 |  |
| 38 | 829.7744 | KFDKALK | 151-157 | 3.46 |  |
| 46 | 903.5229 | Undefined |  | 0.42 |  |

*Fragment sequence calculated based on BLG sequence of <https://www.uniprot.org/uniprotkb/P02754/entry>

**Table 4s.** Classification of the identified tri-hepta peptides according to their contents according

to their basicity and hydrophobicity and relative to the total identified peptides.

| **Neutral**  **or acidic** | **Hydrophobic**  **Basic.** | **Basic** | **Highly**  **basic** |
| --- | --- | --- | --- |
| **FDK**  **DYK**  **EELK**  **NENK**  **EKFDK**  **DTDYK**  **EALEK** | **LPMH**  **CLVR**  **QCLVR**  **DKALK**  **KIIAEK**  **EALEK**  **LPMHIR**  **KFDKALK** | **DYKK**  **CAQK**  **TDYKK**  **DKALK**  **KIIAEK**  **LPMHIR** | **QKK**  **YKK**  **HIR**  **KFDKALK** |
| **3.3%** | **34.8%** | **13.84%** | **37.998%** |
| Basic: **Red** , Hydrophobic: **Green**, Acidic: **Orange,** Neutral: **Black.** The percentage was calculated relative to the total identified tri, tera, pent, hexa and hepta-peptides. | | | |

**Table 5s. Comparison between LCMS and ESI-MS analysis results.**

| **LCMS** | | **ESI-MS** | |
| --- | --- | --- | --- |
| 162 peptides | | 81peptides | |
| Classification by MW:  24 peptides (800-1000)  82 peptides (1000-2000)  37 peptides (2000-3000)  13 peptides (3000-4000)  4 peptides (4000-5000)  2 peptides (> 5000) | | MW (164.8-903.5)  Classification by peptide type:  37 Dipeptides (15%)  Tri-Heptapeptides (84.5%)  7 Tripeptides (17.8%)  14 Tetra peptides (31.6%)  11 Pentapeptides (10.9%)  5 Hexapeptides (19.5%)  3 Heptapeptides (4.5%) | |
| MW |  |  | |
| 800-1000 | 9 basic hydrophobic peptides (38%), 6 neutral hydrophobic peptide and 7 acidic hydrophobic peptides (29%). | Dipeptides | Basic peptides (31.9%)  Hydrophobic peptides (18.9%)  Neutral peptides (47.6%)  Acidic peptides (9.7$) |
| 1000-2000 | 20 basic hydrophobic peptides (24%), 17 neutral hydrophobic peptide and 44 acidic hydrophobic peptides (53%). | Tri-Heptapeptides | Highly basic peptides (37.9%)  Basic peptides (13.84%)  Hydrophobic peptides (34.8%)  Neutral and Acidic peptides (3.3%) |
| 2000-3000 | 9 basic hydrophobic peptides (24%), 6 neutral hydrophobic peptide and 22 acidic hydrophobic peptides (59%). |  |  |
